# A new southern record of the holopelagic annelid *Poeobius meseres* Heath, 1930 (Flabelligeridae)

**DOI:** 10.3897/BDJ.8.e58655

**Published:** 2020-11-30

**Authors:** Charlotte A. Seid, Dhugal J. Lindsay, Greg W. Rouse

**Affiliations:** 1 Scripps Institution of Oceanography, University of California San Diego, La Jolla, CA, United States of America Scripps Institution of Oceanography, University of California San Diego La Jolla, CA United States of America; 2 Japan Agency for Marine-Earth Science and Technology (JAMSTEC), Yokosuka, Japan Japan Agency for Marine-Earth Science and Technology (JAMSTEC) Yokosuka Japan

**Keywords:** polychaete, pelagic, Pacific Ocean, deep sea, COI

## Abstract

**Background:**

The unusual holopelagic annelid *Poeobius meseres* Heath, 1930 (Flabelligeridae) was first collected from Monterey Bay, California and has been subsequently recorded across the northern Pacific from Japan to the Gulf of California. Rare occurrences in the eastern tropical Pacific have extended as far as 7° S off Peru.

**New information:**

Using molecular phylogenetic analysis of a newly-collected specimen from the Salas y Gómez Ridge off Chile, we extend the known geographic range of *P. meseres* southwards by 2040 km. This subtropical specimen showed higher genetic similarity to a specimen from the type locality (< 1.5% pairwise COI distance) than to representatives from the Aleutian Islands and Japan (5-6%), establishing the first genetically-confirmed occurrence of this species in the Southern Hemisphere. The latitudinal range of *P. meseres* encompasses the sole collection locality, off Ecuador, of *Enigma terwielii* Betrem, 1925, a pelagic annelid which has been compared to *P. meseres*, but is indeterminable due to an inadequate description. We therefore suggest that the earlier sole record of *E. terwielii* may have been an occurrence of what is known now as *P. meseres*.

## Introduction

*Poeobius meseres* Heath, 1930 is a holopelagic annelid first collected in a plankton haul at 350 m in Monterey Bay (Heath 1930). These gelatinous, neutrally-buoyant midwater animals are passive detritovores that use a mucous net to capture marine snow (Uttal and Buck 1996). Originally of uncertain taxonomic placement due to the lack of segmentation, chaetae and parapodia, *P. meseres* was later noted to resemble the mostly benthic Flabelligeridae (Hartman 1955) and was only recently confirmed, on the basis of molecular genetics, as belonging to this group (Burnette et al. 2005). Remotely operated vehicles (ROVs) have extensively encountered the species at depths of 25-3975 m at the type locality (Monterey Bay Aquarium Research Institute (MBARI) 2020) and 600-1400 m in Sagami Bay, Japan (Hunt and Lindsay 1999).

**Figure 1.**
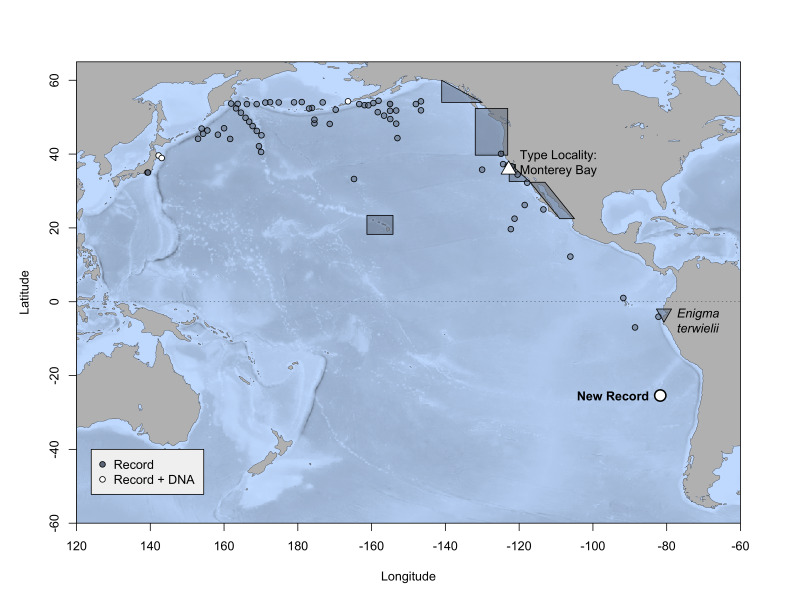
Map of records of *Poeobius meseres* and *Enigma terwielii*. Triangle: type locality. Open symbols: literature records with DNA sequence data available, including this study. Coordinates are listed in Suppl. material 1; records without coordinates are approximated by polygons. The map was generated using the R package marmap (Pante and Simon-Bouhet 2013).

All known occurrences of *P. meseres* are from the Pacific (Fig. 1, Suppl. material 1). Records of collected specimens span the northern Pacific, from Japan to Alaska to California (Pickford 1947, Yamada 1954, Hartman 1955, Berkeley and Berkeley 1960, McGowan 1960, Robbins 1965, Thuesen and Childress 1993, Uttal and Buck 1996, Burnette et al. 2005, Osborn and Rouse 2008, Steinberg et al. 2008, Jimi et al. 2019). ROV video observations include additional occurrences in the Musicians Seamounts (Lindsay et al. 2017), Hawaiian Islands (Monterey Bay Aquarium Research Institute (MBARI) 2020) and Gulf of California (Monterey Bay Aquarium Research Institute (MBARI) 2020). The Ocean Biodiversity Information System (OBIS 2020) and Global Biodiversity Information Facility (GBIF 2020) list no occurrences in the Southern Hemisphere other than this study (Suppl. material 1). Only a few occurrences have been recorded south of Mexico, as far as 6° 58’S, 88° 35’W at 0-1463 m depth, off Peru (McGowan 1960).

These rare eastern tropical Pacific occurrences have been questioned as possibly representing a separate species. McGowan (1960) observed these specimens to have missing or reduced gonads and larger body size than their northern counterparts. He considered the possibility that they represent "a second and hitherto undescribed species with different ecological requirements", but interpreted them as more likely "sterile expatriates from the north," whose reduced gonads reflect a response to unfavourable environmental conditions. Salazar-Vallejo (2008) suggested the presence of cryptic species and called for confirmation of the western Pacific, subtropical and tropical records.

Morphological studies of *P. meseres* are limited, however, by the poor condition of the holotype, the retracted state of the anterior appendages in many specimens and the challenges of sampling fragile midwater organisms (Salazar-Vallejo 2008, Uttal and Buck 1996). Genetic data are therefore of particular importance in assessing the distribution and connectivity of this species. A recent report, comparing *P. meseres* DNA sequences from Japan, the Aleutian Islands and the type locality, interpreted all these records as a single species with a longitudinal distribution of > 4000 km and a maximum intraspecific divergence of approximately 6% (Jimi et al. 2019). No morphological comparisons were made in that study and data available for other annelids suggest this result could be interpreted as representing two species-ranked taxa (Nygren 2014). Here, we report a new southernmost record of *P. meseres*, representing the first genetically-confirmed occurrence of this species in the Southern Hemisphere and establishing a latitudinal distribution of > 8800 km.

## Materials and methods

### Specimen collection

The JAMSTEC DeepTow 6K towed camera system was deployed at station SPG4, southeast of Stockman Guyot on the Salas y Gómez Ridge, ~ 1100 km off Chile, on 6 February 2019 during the R/V *Mirai* cruise MR18-06 Leg 3, “East/Central Pacific International Campaign (EPIC).” A *Poeobius meseres* specimen was collected serendipitously in a passive vertical net affixed to the DeepTow. The tow start and end coordinates were (-25.4062, -81.7417) to (-25.4050, -81.7702) and the maximum depth was 1065 m. Sample collection was conducted under permit Res. Ext. N°45/2018 from SUBPESCA, Chile.

The live specimen was relaxed with 7% MgCl_2_ in fresh water and photographed (Fig. 2a) using a Leica S8Apo stereomicroscope with a Canon EOS Rebel T6i attachment. A posterior tissue sample was minced in RNAlater (Ambion, Austin, TX) and frozen for genetic analysis. The remainder of the specimen was fixed in 10% seawater formalin as a morphological voucher, rinsed with fresh water after at least 24 hours of fixation and transferred to 50% ethanol for long-term archival. The specimen was deposited in the Scripps Institution of Oceanography Benthic Invertebrate Collection (catalogue number SIO-BIC A9529).

Another *P. meseres* specimen was collected on 25 April 2002 from the Japan Trench (38.9350, 143.0933) at a depth of 652 m on dive HPD#0100 of the ROV Hyper-Dolphin, R/V *Kaiyo* cruise KY02-06. The specimen was recorded in HD video *in situ* and photographed in a phototank in the onboard laboratory (Fig. 2b) before being deposited at JAMSTEC (Marine Biological Sample Database, catalogue number 049020).

### DNA sequencing

DNA was extracted using the *Quick*-DNA Microprep Plus Kit (Zymo Research, Irvine, CA and Tustin, CA), following the manufacturer’s protocol. Polymerase chain reaction (PCR) amplification of mitochondrial cytochrome c oxidase subunit I (COI) was performed using the primer pair polyLCO (5’-GAYTATWTTCAACAAATCATAAAGATATTGG-3’) and polyHCO (5’- TAMACTTCWGGGTGACCAAARAATCA-3’) (Carr et al. 2011) in the following reaction: 12.5 μl Apex 2.0x Taq Red DNA Polymerase Master Mix (Genesee Scientific), 1 μl each primer (10 μM), 8.5 μl ddH2O and 2 μl of eluted DNA. The reaction was performed on an Eppendorf thermal cycler using the following temperature profile: 95°C/180 s – (95°C/40 s – 42°C/45 s – 72°C/50 s) * 40 cycles – 72°C/300 s. The PCR product was purified with ExoSAP-IT (USB Corporation, Cleveland, OH). Sanger sequencing was performed by Eurofins Genomics (Louisville, KY) and the consensus sequence was assembled using Geneious v.11.1.5. Sequencing of the Japan Trench specimen was performed using similar methods and reagents.

### Phylogenetic analysis

Newly generated sequences were compared to the three available *P. meseres* COI sequences on GenBank (EU694130.1, LC508299.1, LC508300.1), with *Diplocirrus toyoshioae* Jimi, Fujiwara & Kajihara, 2017 (LC314567.1) and *Brada* sp. (HQ326970.1) as outgroups, based on the flabelligerid phylogeny in Osborn and Rouse (2011). Sequences were aligned using the MAFFT online service v7.471, option L-INS-i (Katoh et al. 2018). Maximum Likelihood analysis was performed with W-IQ-TREE v1.6.11 (Trifinopoulos et al. 2016) using 500 standard bootstrap pseudoreplicates and the ModelFinder option, by which TPM2+F+G4 was chosen as the best-fit model according to the Bayesian Information Criterion. Model-corrected pairwise distances amongst the *P. meseres* sequences were calculated with W-IQ-TREE using the same parameters and HKY+F was chosen as the best-fit model. Uncorrected pairwise distances were calculated using PAUP* v4.0a168 (Swofford 2003) with gaps ignored to account for different sequence lengths. A haplotype network of the *P. meseres* sequences was created with PopART v1.7 (Leigh and Bryant 2015) using the TCS algorithm (Clement et al. 2002).

## Data resources

Sequences were deposited into GenBank: accession numbers MT993561 (SIO-BIC A9529), MT993562 (JAMSTEC 049020).

Phylogenetic data were deposited into TreeBase: http://purl.org/phylo/treebase/phylows/study/TB2:S26923.

## Taxon treatments

### Poeobius meseres

Heath, 1930

9C96A09D-EC16-501E-A7A0-E31739977D0F

#### Materials

- **Type status:** Other material. **Occurrence:** occurrenceDetails: http://api.gbif.org/v1/occurrence/2416968617; catalogNumber: A9529; recordedBy: C. Seid, Miodeli Nogueira Jr., Caitlin Smoot; individualCount: 1; establishmentMeans: NATIVE; occurrenceStatus: PRESENT; preparations: whole (fixed in 10% formalin, preserved in 50% EtOH); otherCatalogNumbers: EP19-0087; occurrenceID: urn:catalog:SIO:BIC:A9529; **Taxon:** scientificName: Poeobius meseres Heath, 1930; higherClassification: Annelida | Polychaeta | Terebellida | Flabelligeridae | Poeobius; kingdom: Animalia; phylum: Annelida; class: Polychaeta; order: Terebellida; family: Flabelligeridae; genus: Poeobius; specificEpithet: meseres; taxonRank: SPECIES; taxonomicStatus: ACCEPTED; **Location:** higherGeography: South America | Chile | | | | Pacific Ocean | |; continent: SOUTH_AMERICA; waterBody: South Pacific Ocean; country: Chile; countryCode: CL; locality: Southeast of Stockman Guyot, Chile; verbatimLocality: South America | Chile | Pacific Ocean | Southeast of Stockman Guyot, Chile; verbatimDepth: 1065 1065 - 0 1065; decimalLatitude: -25.405577; decimalLongitude: -81.755951; geodeticDatum: WGS84; footprintWKT: LINESTRING((-25.406157 -81.74169, -25.404997 -81.770212)); georeferenceVerificationStatus: requires verification; **Identification:** identifiedBy: C. Seid; **Event:** eventDate: 2019-02-06T00:00:00; eventTime: 0804-1110; startDayOfYear: 37; endDayOfYear: 37; year: 2019; month: 2; day: 6; verbatimEventDate: 2/6/2019; fieldNumber: SPG4-DeepTow_20190206; eventRemarks: Cruise MR18-06 Leg 3. Live-sorted material. "Benthic" tow. Data from DeepTow report, reflecting first and last logged coordinates of the entire DeepTow event and the maximum depth reached. Vertical net collects material upon ascent. Maximum depth of 1064 m.; **Record Level:** type: PhysicalObject; modified: 2019-09-17T00:00:00.000+0000; language: en; accessRights: http://vertnet.org/resources/norms.html; institutionID: http://biocol.org/urn:lsid:biocol.org:14844; collectionID: http://grbio.org/cool/t8vy-919z; institutionCode: SIO; collectionCode: BIC; basisOfRecord: PRESERVED_SPECIMEN; dynamicProperties: {"gear":"DeepTow vertical net", "ship":"R/V Mirai" }
- **Type status:** Other material. **Occurrence:** occurrenceDetails: http://api.gbif.org/v1/occurrence/ 2265753314; catalogNumber: 2020019915; occurrenceRemarks: Geodetic datum is unknown but probably Tokyo Datum, WGS72 or WGS84. Thus, the coordinate precision of this data point is increased to 500 m because this is the maximum difference amongst the three datums.; recordNumber: HD100SS1m; individualCount: 1; occurrenceStatus: PRESENT; occurrenceID: urn:catalog:JAMSTEC:Biological Sample Collection:2020019915; **Taxon:** scientificNameID: urn:lsid:marinespecies.org:taxname:330855; scientificName: Poeobius meseres Heath, 1930; kingdom: Animalia; phylum: Annelida; class: Polychaeta; order: Terebellida; family: Flabelligeridae; genus: Poeobius; specificEpithet: meseres; taxonRank: SPECIES; taxonomicStatus: ACCEPTED; **Location:** waterBody: North Western Pacific; country: Japan; countryCode: JP; locality: Japan Trench; decimalLatitude: 38.935; decimalLongitude: 143.0933; geodeticDatum: WGS84; coordinateUncertaintyInMeters: 500; footprintSRS: GEOGCS["GCS_WGS_1984",DATUM["D_WGS_1984",SPHEROID["WGS_1984",6378137,298.257223563]],PRIMEM["Greenwich",0],UNIT["Degree",0.0174532925199433]]; **Event:** eventDate: 2002-04-25T00:00:00; startDayOfYear: 115; endDayOfYear: 115; year: 2002; month: 4; day: 25; fieldNumber: HPD#0100; **Record Level:** modified: 2013-03-18T07:10:17.000+0000; language: en; bibliographicCitation: Marine Biological Sample Database, JAMSTEC; institutionCode: JAMSTEC; collectionCode: Biological Sample Collection; datasetName: Marine Biological Sample Database, JAMSTEC; basisOfRecord: PRESERVED_SPECIMEN

#### Distribution

Northern and south-eastern Pacific Ocean with genetic records from the following localities: United States: Monterey Bay, California (type locality); United States: Aleutian Islands, Alaska; Japan: off Sanriku; Chile: southeast of Stockman Guyot, Salas y Gómez Ridge, south-eastern Pacific (this study).

## Analysis

**Figure 3.**
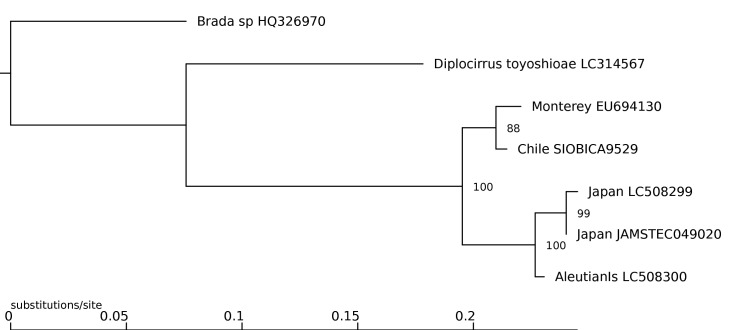
Maximum Likelihood tree of *Poeobius meseres* COI sequences, with *Brada* sp. and *Diplocirrus toyoshioae* as outgroups, based on Osborn and Rouse (2011).

**Figure 4.**
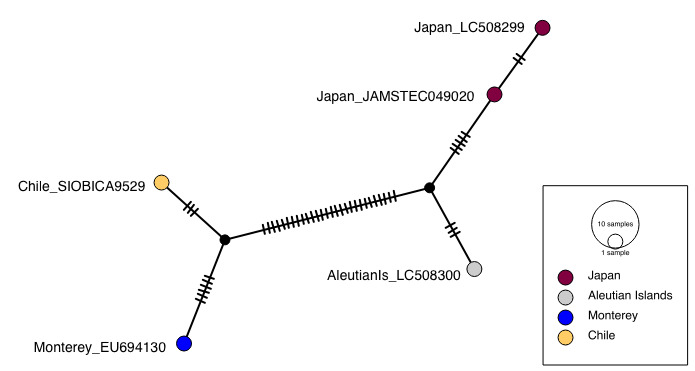
Haplotype network for *Poeobius meseres* COI sequences.

All *Poeobius* sequences formed a strongly supported clade, within which the Chile and Monterey sequences formed a subclade (Fig. 3). The COI sequences of the south-eastern Pacific and Monterey specimens differed by 1.47% (uncorrected; 1.48% corrected) (Table 1), representing 10 base pairs (Fig. 4). This close genetic connection to the type locality supports the identification of the Chile specimen as *P. meseres* sensu stricto. The Chile occurrence thereby extends the confirmed distribution of *P. meseres* by approximately 2040 km south of the previous southern record, establishing a latitudinal range of 80 degrees (54.3° N to 25.4° S) or > 8800 km (Fig. 1). The collection depth of the Chile specimen was consistent with previous eastern tropical Pacific records (Suppl. material 1).

The two sequences from Japan differed from each other by only two base pairs (Fig. 4) and formed a clade with the sequence from the Aleutian Islands (Fig. 3). The south-eastern Pacific sequence differed from those of Japan and the Aleutian Islands by a maximum of 6.44% and 5.29%, respectively (Table 1). This divergence is marginally less than that between the Monterey (EU694130.1) and Japan (LC508299.1) sequences (calculated in this study as 6.46% uncorrected; 6.84% corrected), which were previously accepted as *P. meseres* intraspecific variation by Jimi et al. (2019).

## Discussion

### Distribution of *Poeobius meseres*

The occurrence of *P. meseres* in the oligotrophic waters of South-eastern Pacific Gyre extends the distribution of this species across several broadly defined mesopelagic ecoregions (as described in Sutton et al. (2017)). Additional sampling and imagery, aided by deep submergence technologies, may yield further insight into the global distribution and ecological requirements of this species. The distribution of *P. meseres* in the northern Pacific has been described as largely correlated with the Subarctic Water Mass, although the eastern tropical Pacific occurrences present a notable exception without satisfactory explanation (McGowan 1960). The estimated low population density of *P. meseres* at low latitudes (McGowan 1960), combined with relatively few deep-sea sampling efforts in the south-eastern Pacific, may have contributed to the previous scarcity of *Poeobius* records in the region.

Further assessment of connectivity and cryptic diversity within *Poeobius* will require additional genetic sampling, supported by ecological and morphological observations. For example, an undescribed species of *Poeobius* has been reported from the tropical Atlantic Ocean, 32-998 m (Christiansen et al. 2018), but no DNA sequences are currently available.

### *Enigma terwielii*

The confirmed distribution of *P. meseres* encompasses the sole collection locality, off Ecuador, of the putative flabelligerid *Enigma terwielii* Betrem, 1925. The monotypic *Enigma* is known only from a short preliminary description in Dutch attributed to Betrem (Betrem 1925) and a translation with commentary, stating that the type material has been lost (Hartman 1967). Although the obscure note on *E. terwielii* was not referenced in the original description of *P. meseres*, a subsequent mention of *Enigma* noted its similarities to *Poeobius* (Hartman 1967). Conspicuous shared characteristics, based on the morphological review of *P. meseres* in Salazar-Vallejo (2008), include: absence of parapodia and setae, body embedded in a hyaline mass, papillae on surface epithelium, retractile branchial filaments and palps and coiled intestine.

The type locality of *E. terwielii* is reported as the Bay of Guayaquil, Ecuador (Betrem, pers. comm. in Hartman 1967), at approximately 3° S. A specimen of *Poeobius meseres* was recorded from (-4.0667, -82.2333), only 200 km away (McGowan 1960) and Ecuadorian waters are now well within the extended southern range of *P. meseres*. Although we regard *E. terwielii* as indeterminable given the problems with the description and the absence of type material or figures, we suggest that its sole record was plausibly a southern occurrence of *P. meseres*.

### Conclusion

This study illustrates how even a single specimen, collected by passive and opportunistic use of deep submergence technology, can address a modest, but real gap in fundamental biogeography. Although wide biogeographic distributions are not unusual amongst pelagic annelids and other planktonic organisms (Dales 1957, Dales and Peter 1972, Rouse and Pleijel 2001, Costello et al. 2017), the occurrence records and genetic data required to verify such distributions are often lacking, due to limited sampling (Halanych et al. 2007, Sutton et al. 2017, Levin et al. 2019, Baker and Horton 2020, Drazen et al. 2020). As deep-sea ecosystems face increased threats from deep-sea mining and other anthropogenic impacts (Ramirez-Llodra et al. 2011, Christiansen et al. 2020, Drazen et al. 2020), baseline biodiversity documentation remains important for environmental impact assessments and management efforts.

## Supplementary Material

FB9E3529-8777-5BF9-B186-8B65B8FF4175 10.3897/BDJ.8.e58655.suppl1 Supplementary material 1 Supplementary Table 1 Data type literature summary Brief description Records of *Poeobius meseres* and *Enigma terwielii*: summary of peer-reviewed literature plus additional observations from localities not already represented in the literature. File: oo_468950.xlsx https://binary.pensoft.net/file/468950 Charlotte A. Seid, Dhugal J. Lindsay, Greg W. Rouse

XML Treatment for Poeobius meseres

## Figures and Tables

**Figure 2a.**
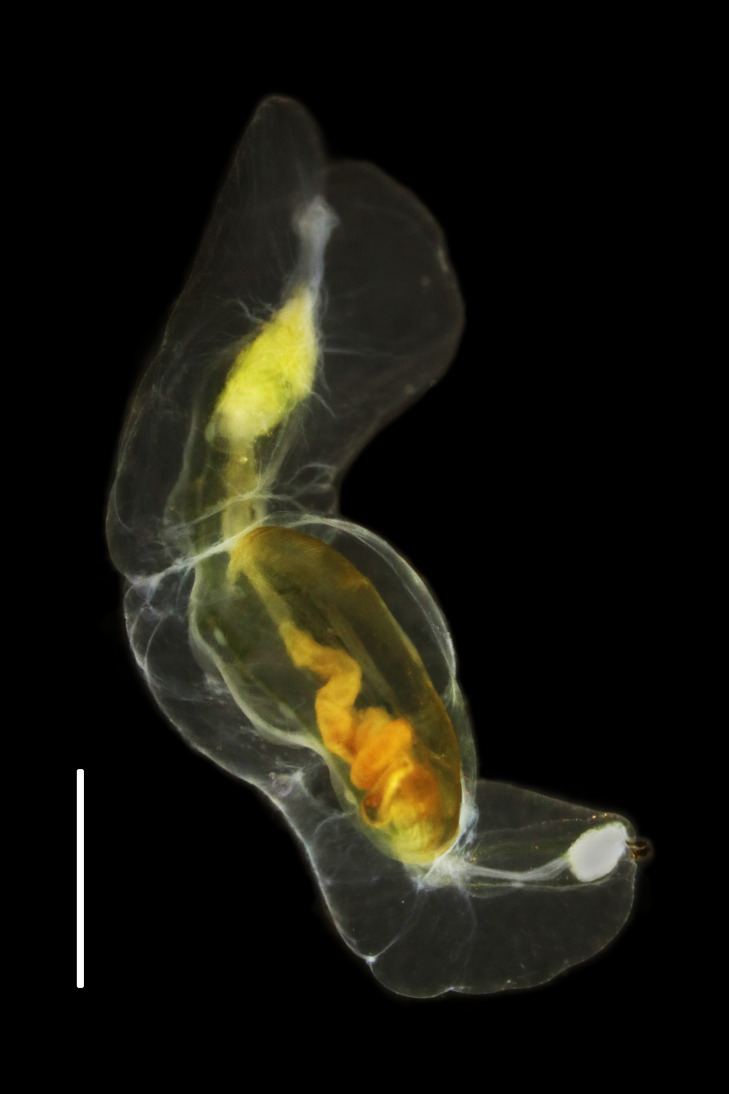
South-eastern Pacific, SIO-BIC A9529; anterior retracted; scale bar: 1 mm.

**Figure 2b.**
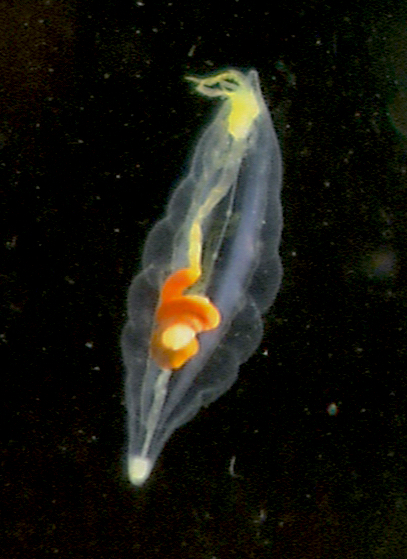
Japan Trench, JAMSTEC 049020; anterior partially retracted.

**Table 1.** Pairwise COI distances between *Poeobius meseres* specimens. New sequences in bold. Model-corrected distances (lower left values): best fit model HKY+F selected via BIC in W-IQ-TREE. Uncorrected (p-) distances (upper right values): PAUP* v4.0a168, gaps ignored.

	**Chile** **(MT993561, SIO-BIC A9529)**	Monterey (EU694130)	Aleutian Islands (LC508300)	Japan (LC508299)	**Japan (MT993562, JAMSTEC 049020)**	
**Chile** **(MT993561, SIO-BIC A9529)**	-	0.014684	0.050798	0.061135	0.054711	Uncorrected
Monterey (EU694130)	0.014792	-	0.055800	0.064611	0.059271	
Aleutian Islands (LC508300)	0.052871	0.058370	-	0.021676	0.012158	
Japan (LC508299)	0.064442	0.068346	0.022198	-	0.003040	
**Japan** **(MT993562, JAMSTEC 049020)**	0.057055	0.062089	0.012294	0.003054	-	
	Corrected					
